# Prenatal levonorgestrel exposure induces autism-like behavior in offspring through ERβ suppression in the amygdala

**DOI:** 10.1186/s13229-017-0159-3

**Published:** 2017-08-17

**Authors:** Yuanlin Zou, Qiaomei Lu, Dan Zheng, Zhigang Chu, Zhaoyu Liu, Haijia Chen, Qiongfang Ruan, Xiaohu Ge, Ziyun Zhang, Xiaoyan Wang, Wenting Lou, Yongjian Huang, Yifei Wang, Xiaodong Huang, Zhengxiang Liu, Weiguo Xie, Yikai Zhou, Paul Yao

**Affiliations:** 1 Internal Medicine of Tongji Hospital, Tongji Medical College, Huazhong University of Science and Technology, Wuhan, 430030 People’s Republic of China; 2 Tongji Wenchang Hospital, Huazhong University of Science and Technology, Wenchang, 571321 People’s Republic of China; 3 Institute of Environmental Medicine, Tongji Medical College, Huazhong University of Science and Technology, Wuhan, 430030 People’s Republic of China; 4 Institute of Burns, Tongren Hospital of Wuhan University, Wuhan, 430060 People’s Republic of China; 5 SALIAI Stem Cell Institute of Guangdong, Guangzhou SALIAI Stem Cell Science and Technology Co. LTD, Guangzhou, 510055 People’s Republic of China

**Keywords:** Amygdala, Autism-like behavior, Estrogen receptor β, Oral contraceptives, Oxidative stress

## Abstract

**Background:**

Autism spectrum disorder (ASD) is characterized by impairments in social communication and restricted or repetitive behaviors or interests. ASD is now diagnosed in more than one out of 100 children and is biased towards males by a ratio of at least 4:1. Many possible explanations and potential causative factors have been reported, such as genetics, sex, and environmental factors, although the detailed mechanisms of ASD remain unclear.

**Methods:**

The dams were exposed through oral contraceptives to either vehicle control (VEH) alone, levonorgestrel (LNG) alone, ethinyl estradiol (EE) alone, or a combination of LNG/EE for 21 days during their pregnancy. The subsequent 10-week-old offspring were used for autism-like behavior testing, and the limbic tissues were isolated for analysis. In another experimental group, 8-week-old male offspring were treated by infusion of ERβ overexpression/knockdown lentivirus in the amygdala, and the offspring were analyzed after 2 weeks.

**Results:**

We show that prenatal exposure of either LNG alone or a LNG/EE combination, but not EE alone, results in suppression of ERβ (estrogen receptor β) and its target genes in the amygdala with autism-like behavior in male offspring, while there is a much smaller effect on female offspring. However, we find that there is no effect on the hippocampus and hypothalamus. Further investigation shows that ERβ suppression is due to LNG-mediated altered methylation on the ERβ promoter and results in tissue damage with oxidative stress and the dysfunction of mitochondria and fatty acid metabolism, which subsequently triggers autism-like behavior. Overexpression of ERβ in the amygdala completely restores LNG-induced ERβ suppression and autism-like behaviors in offspring, while ERβ knockdown mimics this effect, indicating that ERβ expression in the amygdala plays an important role in autism-like behavior development.

**Conclusions:**

We conclude that prenatal levonorgestrel exposure induces autism-like behavior in offspring through ERβ suppression in the amygdala. To our knowledge, this is the first time the potential effect of oral contraceptives on the contribution of autism-like behavior in offspring has been discovered.

**Electronic supplementary material:**

The online version of this article (doi:10.1186/s13229-017-0159-3) contains supplementary material, which is available to authorized users.

## Background

Autism spectrum disorder (ASD) is characterized by impairments in social communication and restricted or repetitive behaviors or interests [1]. ASD is now diagnosed in more than one out of 100 children [2]. The identified prevalence of ASD has increased in a short period of time with an epidemic proportion in the world. The detailed mechanism of ASD remains unclear [3], and many possible explanations and potential causative factors have been reported. A three-hit theory, which includes the combination of genetic/epigenetic, environmental, and sex factors for the mechanism of ASD, has been widely accepted [4]. Interestingly, ASD is biased towards males by a ratio of at least 4:1, suggesting that fetal or perinatal exposure to elevated male hormones may increase susceptibility towards autism [5–7]. On the other hand, there is a hypothesis that estrogen, especially ethinyl estradiol (EE) from COC (combined oral contraceptives), may play a role in ASD development [8], although current literatures have not reached a conclusion [9].

ERβ (estrogen receptor β) is the principal estrogen receptor expressed in brain areas [3, 10, 11]. An earlier study has found a significant association of the ERβ gene with scores on the Autism Spectrum Quotient and the Empathy Quotient in ASD subjects, and in the ERβ gene, the C allele in rs1271572 and rs1152582 were associated with higher AQ in the typical population and were also found to be more frequent in cases than in controls [12]. Moreover, ERβ mediates some of the effects on anxiety, locomotor activity, fear responses, and learning behavior in mice [13]. It has been recently reported that the ERβ expression in the middle frontal gyrus of ASD subjects is decreased, indicating that ERβ expression in the brain may play a role in the development of ASD [14].

We have previously reported that ERβ is responsible for the basal expression of superoxide dismutase (SOD2) [15] and estrogen-related receptor α (ERRα) [16], where ERRα expression regulates mitochondrial function and fatty acid metabolism and SOD2 regulates oxidative stress [17, 18]. Therefore, ERβ suppression may bring potential damage and disorders to the brain tissues through SOD2 suppression-mediated oxidative stress and DNA break as well as ERRα suppression-mediated dysfunction of mitochondria and fatty acid metabolism.

Oral contraceptive hormones, including EE and levonorgestrel (LNG), have been the mainstay contraceptive hormones for the last four decades and have potential effects on behavior and the central nervous system [19]. We hypothesize that prenatal exposure of oral contraceptive hormones may contribute to ASD development in offspring. Previous studies demonstrate that 10 μg/rat/day EE and 20 μg/rat/day LNG are the lowest individual doses found to inhibit ovulation in intact rodents [20–23]. In order to find the best dose for the treatment of our dams, we tried two combination doses, with the low dose of 10 μg EE/20 μg LNG and high dose of 30 μg EE/60 μg LNG per rat each day, as recommended by a recent study [19]. Our preliminary data showed that high doses brought many problems to the dams, including abnormal pregnancy, such as bleeding, or, in some case, the pregnancy was prevented or interrupted even though a vaginal plug was found after mating. Therefore, we chose the dosage of 10 μg EE/20 μg to be the final treatment for the dams.

In this study, we show that prenatal exposure of either LNG alone or combined LNG/EE treatment induces significant ERβ suppression in the amygdala and autism-like behavior in offspring, while EE2 treatment alone shows no effect. Further investigation shows that ERβ suppression is due to LNG-mediated altered methylation on the ERβ promoter. Overexpression of ERβ in the amygdala completely restores LNG-mediated autism-like behavior in offspring, while ERβ knockdown mimics this effect. This suggests that prenatal LNG exposure may play a dominant role in oral contraceptive-mediated autism-like behavior through ERβ suppression in the amygdala.

## Methods

### Materials

The antibodies for ERRα (ab37438), H2AX (ab20669) and γH2AX (ab2893), H3K9me2 (ab1220), and H3K9me3 (ab8898) and H3K27me3 (ab6002) were obtained from Abcam. Antibodies for β-actin (sc-47,778), ERβ (sc-137,381), and SOD2 (sc-30,080) were obtained from Santa Cruz Biotechnology. LNG and EE were obtained from Sigma. 3-Nitrotyrosine (3-NT) was measured using the 3-Nitrotyrosine ELISA Kit (ab116691 from Abcam) per manufacturers’ instructions. The mitochondrial fraction was isolated using a Pierce Mitochondria Isolation Kit (Pierce Biotechnology) per manufacturers’ instructions. Nuclear extracts were prepared using the NE-PER Nuclear and Cytoplasmic Extraction Reagents Kit (Pierce Biotechnology). Protein concentration was measured using the Coomassie Protein Assay Kit (Pierce Biotechnology).

### Generation of lentivirus

**Rat ERβ expression lentivirus.** The rat complementary DNA (cDNA) for ERβ was obtained from Open Biosystems. The cDNA for rat ERβ was subcloned into the pLVX-Puro vector (from Clontech) with the restriction sites of Xho1 and Xba1 using the below primers: ERβ forward primer: 5′-gtac-**ctcgag**-atg aca ttc tac agtcctgct-3′ (Xho1) and ERβ reverse primer: 5′-gtac-**tctaga**-tcactgagactg tag gttctg-3′ (Xba1). The ERβ or empty control (CTL) was expressed through Lenti-X™ Lentiviral Expression Systems (from Clontech) per manufacturers’ instructions.

**Rat ERβ shRNA lentivirus.** According to our preliminary data from in vitro cell culture experiments, the following sequence was confirmed as the most effective in knockdown rat ERβ: 5′-GGT CAT GTG AAG GAT GTA AGG-3′. The shRNA template for ERβ or scrambled was designed (sense strand + loop + antisense strand), and the related double strand DNA (dsDNA) was synthesized and annealed. They were then inserted into the pLVX-shRNA1 vector (from Clontech) using BamH1/EcoR1 restriction sites. The Scrambled (CTL) or shERβ lentivirus was then expressed through Lenti-X™ shRNA Expression Systems (from Clontech) per manufacturers’ instructions.

### In vivo rat experiments

Sprague–Dawley rats (Wuhan, China) were maintained under standard 12-h light/dark cycles and given ad libitum access to food and water. The animal protocol conformed to the US NIH guidelines (Guide for the Care and Use of Laboratory Animals, No. 85–23, revised 1996) and was reviewed and approved by the Institutional Animal Care and Use Committee from Tongji Medical College and Wuhan University.

#### Rat protocol 1

Adult (3 months old) female Sprague–Dawley rats were monitored for estrous cycles with daily vaginal smears. Only rats with at least two regular 4- to 5-day estrous cycles were included in the studies. The females were caged with proven males, and pregnancy was verified by observation of a sperm plug, which was designated as day 0 of pregnancy. Dams were randomly assigned to the following drug conditions: LNG (20 μg levonorgestrel), EE (10 μg ethinyl estradiol), or LNG/EE (20 μg LNG and 10 μg EE combination), and VEH rats received the same volume of vehicle. Drugs were suspended in 5% ethanol in organic sesame oil, and 0.1 ml were given daily through subcutaneous injection at the nape starting from day 1 until pup delivery for ~21 days. The same individuals handled the rats for injections, and experimenters were naive to drug conditions. The male and female offspring were separated from the dams on day 21 and fed until 9–10 weeks old for further experiments. Parts of 9- to 10-week-old offspring were then used for behavior tests, including autism-like and anxiety-like behavior testing. After that, the offspring were sacrificed, and the different brain tissues, including the amygdala, hypothalamus, and hippocampus, were isolated, flash frozen in dry ice, and then stored in a −80 °C freezer for the analysis of gene expression, SOD2 activity, superoxide anion release, DNA damage, mitochondrial function, and fatty acid metabolism. In addition, other parts of the offspring were used for the isolation of primary amygdala neurons.

#### Rat protocol 2

The male offspring (8 weeks old) from the VEH and LNG groups in *rat protocol 1* were anesthetized with a mixture of ketamine (90 mg/kg) and xylazine (2.7 mg/kg) and implanted with a guide cannula targeting the amygdala (26 gauge; Plastics One). The following coordinates were chosen for the amygdala: −2.0 mm posterior to the bregma, ±4.2 mm from the midline, and −7.2 mm from the skull surface on which it was based. Cannula was attached to the skull with dental acrylic and jeweler’s screws and closed with an obturator [24]. An osmotic minipump (Alzet model 2002; flow rate 0.5 μl/h; Cupertino, CA) connected to a 26-gauge internal cannula that extended 1 mm below the guide was implanted and used to deliver ERβ overexpression (↑ERβ), ERβ knockdown (shERβ), or empty (EMP) lentivirus. Vehicle consisting of artificial cerebrospinal fluid (aCSF; 140 mM NaCl, 3 mM KCl, 1.2 mM Na_2_HPO_4_, 1 mM MgCl_2_, 0.27 mM NaH_2_PO_4_, 1.2 mM CaCl_2_, and 7.2 mM dextrose, pH 7.4) was used for the infusion of the lentivirus. Infusion (flow rate 0.5 μl/h) begun immediately after placement of the minipump. 0.5 μl of total 2 × 10^3^ cfu of lentivirus was infused for 1 h. Rats received the infusion of lentivirus to either knockdown or overexpress ERβ. The experimental rats were separated into four groups (10 per group): group 1, VEH offspring with empty control lentivirus infusion (VEH/EMP); group 2, LNG offspring with empty control lentivirus infusion (LNG/EMP); group 3, LNG offspring with ERβ expression lentivirus infusion (LNG/↑ERβ); and group 4, VEH offspring with ERβ knockdown lentivirus infusion (VEH/shERβ); cannula placement was verified histologically postmortem by the injection of 0.5 μl of India ink (volume matched drug delivery in the experiments). Rats whose dye injections were not located in the amygdala were excluded from the data analysis. Two weeks after the lentivirus infusion, the offspring were used for behavior tests followed by biomedical analysis, as indicated in rat protocol 1.

### Animal behavior test

The animal behavior test of offspring was carried out at 10 weeks of age. Female offspring were tested in the diestrus phase, which was confirmed by vaginal smears. Autism-like behavior was evaluated using the marbles burying test (MBT), social interaction (SI) test, elevated plus maze (EPM), and open-field test [25, 26].

#### Marbles burying test

In brief, each rat is placed in a clean cage (35 × 23 × 19 cm^3^) filled with wood chip bedding to a depth of 5 cm containing 20 colored glass marbles (1 cm diameter) placed in a 5 × 4 arrangement. The number of marbles buried (>50% covered by bedding material) in 30 min was hand-scored by the experimenter [25, 27].

#### Social interaction test

In short, the subjects (test and stranger) were separately habituated to the arena for 5 min before the test. During each test, the rats were placed into the apparatus over a period of 20 min and the time spent following, mounting, grooming, and sniffing any body parts of the other rat was taken as an indicator of social engagement, and was hand-scored by the experimenter. The animal used as the “stranger” was used only once and was a Sprague–Dawley rat of the same gender, weight, and age, with no previous contact with the test rats [25, 27].

#### Elevated plus maze

All behavioral tests were performed when the rats were 10 weeks old. To investigate the presence of anxiety-like behavior in male and female offspring, the EPM test, a well-established rodent model used to characterize anxiety-like behavior, was performed. The Elevated Plus Maze Package with IR Beam Detection for Rat (Cat #: MED-ELVM-1R) was obtained from Med Associates Inc. The maze is comprised of two open and two closed arms. Dual sensors at the entrance to each goal runway allow software to differentiate between runway exploration and entrance, resulting in more accurate position detection. The rats were placed in the junction area, and their movements were measured for 5 min using infrared beams installed on each arm and automatically registered by the MED-PC software (Cat #: SOF-735, Med Associates) for further analysis [24].

#### Open-field test

Immediately following the EPM, the rats were tested in an open-field arena using the Open Field Starter Package for Rat (Cat #: MED-OFA-RSU, Med Associates Inc.). The open-field arena consists of fixed position walls mounted to the system base. Animal movement is tracked using three of 16 beam IR arrays. IR beams are located on both the *X* and *Y* axes for positional tracking and the *Z* axis for rearing detection. The animals were placed in the center of an open field, and exploration was assessed for 30 min. The dimensions of the arena were 40 cm × 40 cm, of which the peripheral 10 cm were considered the peripheral zone and the central 20 cm were considered the central zone [24].

### In vitro primary culture of amygdala neurons

Ten-week-old offspring were used for the preparation of primary amygdala neurons. Amygdala tissues were dissected from rat offspring that were humanely sacrificed through cervical dislocation. The tissues were treated with 0.05% trypsin EDTA for 15 min at 37 °C. Trypsin EDTA was replaced with soybean trypsin inhibitor (Sigma) for 5 min at 37 °C to stop the reaction. This was then replaced with supplemented Neurobasal A (Invitrogen) followed by mechanical dissociation. The cells were then resuspended in culture media, including Neurobasal A, B27, 1×GlutaMAX and 100 U/ml Pen/Strep (from Invitrogen), and then, the cells were incubated at 37 °C, 5% CO_2_ [28]. The isolated amygdala neurons were used for the analysis of DNA methylation, epigenetic changes by chromatin immunosuppression (ChIP) assay on the ERβ promoter, and in vitro fatty acid lipid uptake.

### RT reaction and real-time quantitative PCR

Total RNA from the amygdala was extracted using the RNeasy Micro Kit (Qiagen), and the RNA was reverse-transcribed using an Omniscript RT kit (Qiagen). All the primers were designed using Primer 3 Plus software with the Tm at 60 °C, the primer size of 21 bp, and the product length in the range of 140–160 bp (see Additional file 1: Table S1). The primers were validated with the amplification efficiency in the range of 1.9–2.1, and the amplified products were confirmed with agarose gel. The real-time quantitative PCR was run on iCycleriQ (Bio-Rad) with the Quantitect SYBR green PCR kit (Qiagen). The PCR was performed by denaturation at 95 °C for 8 min, followed by 45 cycles of denaturation at 95 °C, annealing at 60 °C, and extension at 72 °C for 10 s, respectively. One microliter of each cDNA was used to measure target genes. The β-actin was used as the housekeeping gene for transcript normalization, and the mean values were used to calculate relative transcript levels with the ^ΔΔ^CT method per instructions from Qiagen. In brief, the amplified transcripts were quantified by the comparative threshold cycle method using β-actin as a normalizer. Fold changes in gene messenger RNA (mRNA) expression were calculated as 2^−ΔΔCT^ with CT = threshold cycle, ΔCT = CT (target gene)-CT (β-actin), and the ΔΔCT = ΔCT (experimental)-ΔCT (reference).

### Western blotting

The amygdala tissues were lysed in an ice-cold lysis buffer (0.137 M NaCl, 2 mM EDTA, 10% glycerol, 1% NP-40, 20 mM Tris base, pH 8.0) with protease inhibitor cocktail (Sigma). The proteins were separated in 10% SDS-PAGE and further transferred to the PVDF membrane. The membrane was incubated with appropriate antibodies and washed and incubated with HRP-labeled secondary antibodies, and then, the blots were visualized using the ECL+plus Western Blotting Detection System (Amersham). The blots were quantitated by IMAGEQUANT, and the results were normalized by β-actin.

### SOD2 activity assay

The SOD2 was obtained from the mitochondrial fraction that was isolated using a Pierce Mitochondria Isolation Kit (Pierce) per manufacturers’ instructions. The SOD activity was measured as described previously [29]. In brief, a stable O_2_^−^ source was generated through the conversion action of XOD (xanthine oxidase) from xanthine and was mixed with chemiluminescent (CL) reagents to achieve a stable light emission. The SOD2 sample injection can scavenge O_2_^−^, and the subsequent decrease of chemiluminescent response is proportional to SOD2 activity. This system can have a detection limit of 0.001 U ml^−1^ with the linear range of 0.03 ~ 2.00 U ml^−1^. The results were normalized by protein concentration and were expressed as units per milligram proteins (U/mg).

### In vivo superoxide anion (O_2_^−^) release

Superoxide anion release from the amygdala tissue was determined by a luminol-EDTA-Fe enhanced chemiluminescence (CL) system supplemented with DMSO-TBAC (dimethyl sulfoxide-tetrabutyl ammonium chloride) solution for extraction of released O_2_^−^ from tissues, as described previously. The superoxide levels were calculated from the standard curve generated by the xanthine/xanthine oxidase reaction [30].

### Measurement of DNA breaks

The 8-OHdG formation was measured using an OxiSelect™ Oxidative DNA Damage ELISA Kit (Cat No. STA320, from Cell Biolabs Inc.) per manufacturers’ instructions. The formation of γH2AX was measured from nuclear extracts by western blotting using H2AX as the input control.

### Evaluation of mitochondrial function

*Mitochondrial DNA copies.* The genomic DNA was extracted from the amygdala tissue using a QIAamp DNA Mini Kit (Qiagen), and the mitochondrial DNA was extracted using the REPLI-g Mitochondrial DNA Kit (Qiagen). The purified DNA was used for the analysis of genomic β-actin (marker of the nuclear gene) and ATP6 (ATP synthase F0 subunit 6, marker of the mitochondrial gene), respectively, using the qPCR method mentioned above. The primers for genomic β-actin: forward 5′-acc aca gct gag agg gaa atc-3′ and reverse 5′-att gcc gat agt gat gac ctg-3′. The primers for ATP6 are forward 5′- tag ggc ttc ttc ccc ata cat-3′ and reverse 5′-tta gtg aga tgg ggg ttc ctt-3′. The mitochondrial DNA copies were obtained from relative ATP6 copies that were normalized by β-actin copies using the ^ΔΔ^CT method.

*Intracellular ATP level.* The intracellular ATP level was determined using the luciferin-/luciferase-induced bioluminescence system. An ATP standard curve was generated at concentrations of 10^−12^ to 10^−3^ M. Intracellular ATP levels were calculated and expressed as nanomole per milligram protein [30].

### DNA methylation analysis

We developed a real-time PCR-based method for methylation-specific PCR (MSP) analysis on the rat ERβ promoter according to the previously described method with some modifications [31–33]. The rat genomic DNA from the amygdala was extracted and purified and then treated by bisulfite modification using the EpiJET Bisulfite Conversion Kit (#K1461, Fisher). The modified DNA was then amplified using methylated and unmethylated primers for MSP that were designed using the Methprimer software (http://www.urogene.org/cgi-bin/methprimer/methprimer.cgi) with the below details: methylated primer forward 5′-TTT TTT TTA GGT TTT TAA AAG ACG T-3′, reverse 5′-ATA CCA ATA ACA ACA CCA ACC G-3′, unmethylated primer forward 5′-TTT TTT TTA GGT TTT TAA AAG ATG T-3′, and reverse 5′-AAT ACC AAT AAC AAC ACC AAC CAC T-3′, the product size 194 bp (methylated) and 195 bp (unmethylated), CpG island size 134 bp, and Tm 68–70 °C. The final methylation readout was normalized by unmethylated input PCR, the PCR products were confirmed by electrophorese using 2% agarose gel, and the DNA bands were imaged.

### Chromatin immunoprecipitation

Cells were washed and crosslinked using 1% formaldehyde for 20 min and terminated by 0.1 M glycine. Cell lysates were sonicated and centrifuged. Five hundred micrograms of protein were pre-cleared by BSA/salmon sperm DNA with preimmune IgG and a slurry of protein A agarose beads. Immunoprecipitations were performed with the indicated antibodies, BSA/salmon sperm DNA, and a 50% slurry of protein A agarose beads. Input and immunoprecipitates were washed and eluted, then incubated with 0.2 mg/ml proteinase K for 2 h at 42 °C, followed by 6 h at 65 °C to reverse the formaldehyde crosslinking. DNA fragments were recovered by phenol/chloroform extraction and ethanol precipitation. A 140 bp fragment in the range of −200~0 from the transcription start site on the rat ERβ promoter was amplified by real-time PCR (qPCR) using the below primers: forward 5′-ggg tgt ccc tag tgg atg act-3′ and reverse 5′-aaa aga gtg tgg gag ggt agc-3′.

### Evaluation of fatty acid metabolism

In vitro *lipid transport assay*. Primary amygdala neurons were seeded in a 12-well plate and grew until they were 80% confluent. After treatment, 0.5 mCi well^−1^ of ^14^C oleic acid (OA) from PerkinElmer was added. After 4 h of incubation, the cells were washed and harvested, and the total radioactivity was quantitated by scintillation counting [34].

*Rate of fatty acid oxidation from tissues*. The fatty acid oxidation (FAO) rate was measured by evaluation of palmitate oxidation using published methods with minor modifications [35, 36]. In brief, the amygdala tissue was homogenized, and 30 μl of tissue homogenate were then incubated in 370 μl of DMEM containing 0.5% BSA/0.2 mM palmitate/0.5 μCi/ml 1-^14^C-palmitate at 37 °C for 2 h. The incubation was stopped by the injection of 0.2 ml of 40% perchloric acid into the tube to acidify the medium and liberate the CO_2_. The CO_2_ was trapped by a filter paper saturated with 20 μL of 1 M NaOH located on the top of the cap. After overnight isotopic equilibration at room temperature, the filter was removed, and the trapped ^14^CO_2_ and ^14^C acid-soluble products generated by the oxidation of [^14^C] palmitate were counted to calculate the total palmitate oxidation. The protein concentrations were measured, and the results were expressed as nanomole per milligram proteins per hour (nmol/mg/h).

### Statistical analysis

The data was given as mean ± SEM, and all the experiments were performed at least in quadruplicate unless indicated otherwise. In order to evaluate the effects of different hormonal treatments on the examined parameters in both males and females, the two-way analysis of variance (ANOVA) followed by the Bonferroni post hoc test was used; to evaluate the effects of hormonal treatments on the examined parameters in either males or females only, the one-way ANOVA followed by the Tukey−Kramer test was used to determine statistical significance of different groups by SPSS 22 software, and a *P* value of <0.05 was considered significant.

## Results

### Prenatal levonorgestrel exposure suppresses ERβ expression and its target genes in the amygdala in male offspring (10 weeks old) and has less of an effect on female offspring

**Fig. 1:**
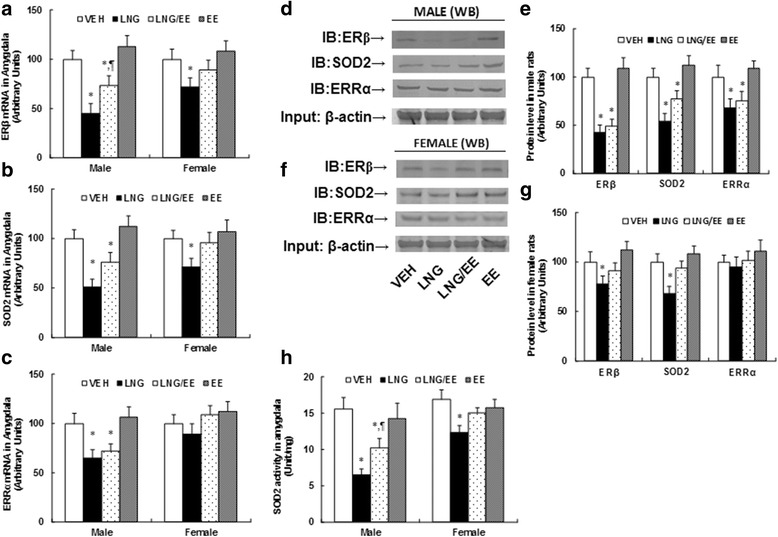
Prenatal levonorgestrel exposure suppresses ERβ expression and its target genes in the amygdala in male offspring (10 weeks old) and has less of an effect in females. Three-month-old pregnant dams were exposed to LNG (20 μg levonorgestrel), EE (10 μg ethinyl estradiol), LNG/EE (20 μg LNG and 10 μg EE combination), or VEH (vehicle, 5% ethanol in organic sesame oil) by subcutaneous daily injection of 0.1 ml for 21 days until pup delivery. Both male and female offspring were sacrificed at 10 weeks of age to isolate the amygdala for further analysis. mRNA levels in the amygdala for genes of **a** ERβ, **b** SOD2, and **c** ERRα, *n* = 5. **d** The representative pictures of protein levels of male offspring by western blotting. **e** Protein expression quantitation for **d**, *n* = 5. **f** The representative pictures for protein levels of female offspring by western blotting. **g** Protein expression quantitation for **f**, *n* = 5. **h** SOD2 activities for both male and female offspring in the amygdala, *n* = 6. *P < 0.05 vs. VEH group; *P* < 0.05 vs. LNG group. Results are expressed as mean ± SEM

We first evaluated the effect of prenatal exposure of COC on the expression of ERβ and its target genes in the limbic system in the offspring. Three-month-old pregnant dams were exposed to LNG (20 μg levonorgestrel), EE (10 μg ethinyl estradiol), LNG/EE (20 μg LNG and 10 μg EE combination), or VEH (the same volume of vehicle) through subcutaneous daily injections of 0.1 ml for 21 days until pup delivery. The limbic system, including the hypothalamus, hippocampus, and amygdala, were isolated for the analysis of gene expression. In Additional file 1: Table S2, we measured the gene expression of AR (androgen receptor), ERα (estrogen receptor α), and GPER1 (G protein-coupled estrogen receptor-1) from the hypothalamus, hippocampus, and amygdala, the brain areas known to be involved in the regulation of mood behavior. The results showed that there was no difference in expression in either male or female offspring. We also measured the gene expression of ERβ and its target genes, including SOD2 and ERRα in the hypothalamus and hippocampus from both male and female offspring (see Additional file 1: Figure S1), and found that there was no difference in those genes. We then measured the mRNA expression of ERβ (see Fig. 1a) and its target genes SOD2 (see Fig. 1b) and ERRα (see Fig. 1c) in the amygdala. We found that treatment of either LNG alone or LNG/EE combination significantly suppressed the expression of ERβ, SOD2, and ERRα in male offspring, and EE alone had no effect. On the other hand, in female offspring, LNG alone could still significantly suppress these genes but had a much smaller effect compared to that in male offspring, and EE alone and LNG/EE combination had no effect. We then measured the protein levels in the amygdala from both male and female offspring. The results showed that protein levels of ERβ and its target genes SOD2 and ERRα were suppressed significantly in male offspring by treatment of LNG alone and LNG/EE combination, and EE alone had no effect (see Fig. 1d, e). On the other hand, there was much less of an effect in female offspring. LNG alone slightly decreased protein expression, while LNG/EE combination and EE alone showed no effect on protein expression (see Fig. 1f, g). We finally measured the SOD2 activity (see Fig. 1h), and similar patterns, like SOD2 protein expression, were observed. SOD2 activity decreased significantly in LNG and LNG/EE treatment in male offspring, while in female offspring, only LNG alone slightly decreased activity, and LNG/EE and EE treatment showed no effect (see detailed statistical information in Additional file 1: Data S1). Our results suggest that LNG may play a dominant role in prenatal exposure of COC-induced ERβ suppression in the amygdala in offspring, while EE seems to have no effect, and that females are less responsive to prenatal LNG exposure, compared to male offspring.

### Prenatal levonorgestrel exposure induces ERβ suppression in the amygdala through increased methylation on the ERβ promoter, while EE has no effect

**Fig. 2:**
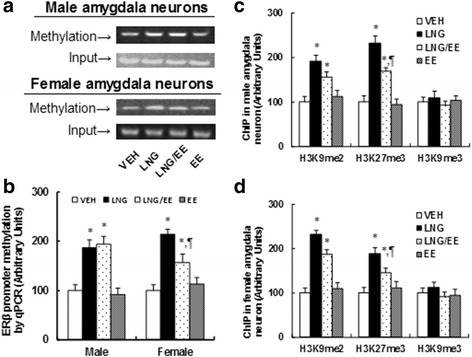
Prenatal levonorgestrel exposure induces ERβ suppression in amygdala neurons through increased methylation on the ERβ promoter, while EE has no effect. **a** The amygdala neurons were isolated from 10-week-old male/female offspring for in vitro cell culture analysis. The representative bands for ERβ methylation in amygdala neurons from both male (*upper panel*) and female (*lower panel*) offspring. **b** DNA methylation on ERβ by real-time PCR-based methylation-specific PCR (MSP) analysis in amygdala neurons, *n* = 5. **c** ChIP analysis on the ERβ promoter in male amygdala neurons, *n* = 5. **d** ChIP analysis on ERβ promoter in female amygdala neurons, *n* = 5. *P < 0.05 vs. VEH group; *P* < 0.05 vs. LNG group. Results are expressed as mean ± SEM

We measured DNA methylation on the ERβ promoter using methylation-specific PCR (MSP) analysis. In Fig. 2a, b, both LNG and LNG/EE treatments significantly increased DNA methylation on the ERβ promoter in amygdala neurons from both male and female offspring (10 weeks old), while EE treatment had no effect. On the other hand, there was much less of an effect on DNA methylation with the LNG/EE treatment in females compared in males. We then measured the epigenetic changes on the ERβ promoter using ChIP techniques. The results showed significantly increased H3K9 di-methylation (H3K9me2) and H3K27 tri-methylation (H3K27me3) on the ERβ promoter from both male (see Fig. 2c) and female offspring (see Fig. 2d) with LNG alone and LNG/EE combination treatment, while EE had no effect (see detailed statistical information in Additional file 1: Data S2). Our results indicate that prenatal levonorgestrel exposure-induced ERβ suppression in amygdala neurons is due to increased DNA methylation on the ERβ promoter.

### Prenatal levonorgestrel exposure induces ROS generation and DNA damage, along with dysfunction of mitochondria and fatty acid metabolism in the amygdala in male offspring (10 weeks old), while there is less of an effect in female offspring

**Fig. 3:**
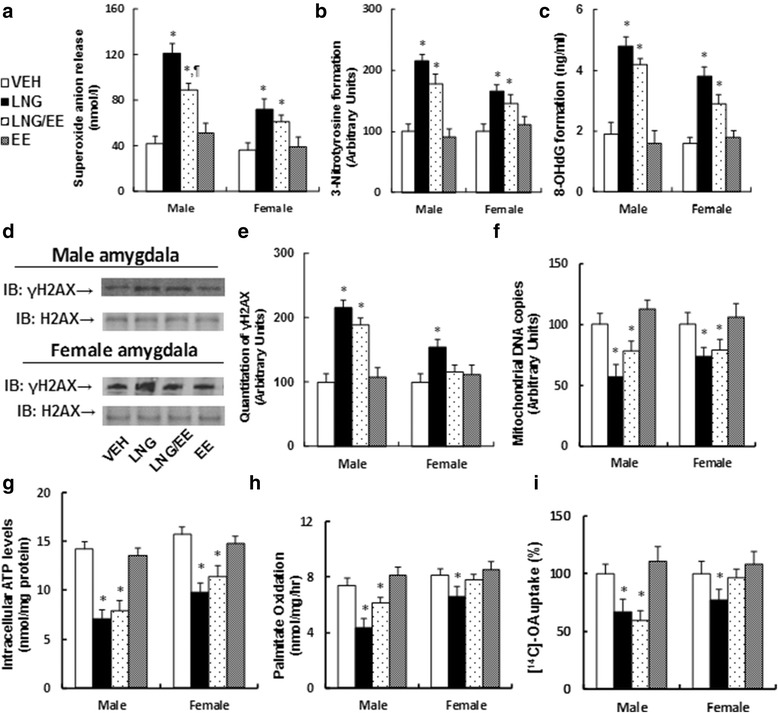
Prenatal levonorgestrel exposure induces ROS generation and DNA damage and dysfunction of mitochondria and fatty acid metabolism from the amygdala in male offspring (10 weeks old), while there is less of an effect in females. **a**–**h** The amygdala tissues were isolated from 10-week-old male/female offspring for further analysis. **a** In vivo superoxide anion release, *n* = 6. **b** Quantitation of 3-nitrotyrosine (3-NT) formation, *n* = 5. **c** 8-OHdG formation, *n* = 5. **d** Representative γH2AX western blotting band for both male (*upper panel*) and female (*down panel*) offspring. **e** Quantitation of γH2AX formation for **d**, *n* = 5. **f** Mitochondrial DNA copies, *n* = 4. **g** Intracellular ATP levels, *n* = 5. **h** The in vivo palmitate oxidation rate, *n* = 5. **i** The amygdala neurons were isolated from 10-week-old male/female offspring for in vitro ^14^C–OA fatty acid uptake, *n* = 5. *P < 0.05 vs. VEH group; *P* < 0.05 vs. LNG group. Results are expressed as mean ± SEM

We evaluated the molecular consequences of prenatal levonorgestrel exposure-induced ERβ suppression in the amygdala in both male and female offspring. We showed that prenatal levonorgestrel exposure (both LNG and LNG/EE treatment) significantly increased superoxide anion (O_2_^−^) release from amygdala tissues (see Fig. 3a), 3-nitrotyrosine formation (see Fig. 3b), 8-OHdG formation (see Fig. 3c), and DNA damage with γH2AX formation (see Fig. 3d, e); it also suppressed mitochondrial function, including decreased mitochondrial DNA copies (see Fig. 3f) and intracellular ATP level (see Fig. 3g). Furthermore, it suppresses fatty acid metabolism, including decreased in vivo fatty acid oxidation (see Fig. 3h) and in vitro fatty acid uptake (see Fig. 3i). Interestingly, there was much less damage in the amygdala in female offspring than in male offspring, which is consistent with the ERβ expression in the amygdala (see detailed statistical information in Additional file 1: Data S3). These results indicate that prenatal levonorgestrel exposure-induced ERβ suppression in both male and female offspring (10 months old) may contribute to LNG-mediated molecular damage in the amygdala.

### Prenatal levonorgestrel exposure induces autism-like behavior in male offspring (10 weeks old), while there is less of an effect in female offspring

**Fig. 4:**
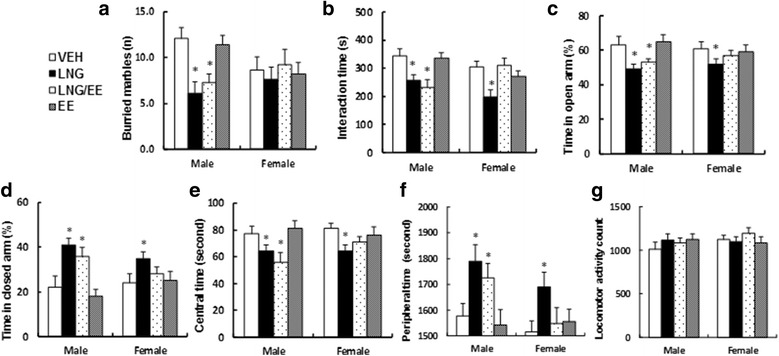
Prenatal levonorgestrel exposure induces autism-like behavior in male offspring (10 weeks old), while there is less of an effect in females. Three-month-old pregnant dams were exposed to LNG (20 μg levonorgestrel), EE (10 μg ethinyl estradiol), LNG/EE (20 μg LNG and 10 μg EE combination), or VEH (vehicle, 5% ethanol in organic sesame oil) through 0.1 ml of subcutaneous daily injection for 21 days until pup delivery. Both male and female offspring were used for autism-like behavior tests at 10 weeks old. **a** Buried marbles tests, *n* = 8. **b** Interaction time, *n* = 8. Time spent in **c** open and **d** closed arms in EPM test, *n* = 8. Time spent in **e** central and **f** peripheral area and **g** locomotor activity in the open-field test, *n* = 9. *P < 0.05 vs. VEH group. Results are expressed as mean ± SEM

We measured the effect of prenatal levonorgestrel exposure on autism-like behavior in offspring. The male offspring that received the LNG and LNG/EE treatment had significant less buried marbles (see Fig. 4a) and interaction time (see Fig. 4b) and spent significantly less time in the open arm (see Fig. 4c) and more time in the closed arm (see Fig. 4d) in the EPM (elevated plus maze) test compared to the vehicle-treated animals (VEH). Also, the male offspring spent less time in the central area (see Fig. 4e) and more time in the peripheral area (see Fig. 4f) but without changes in total horizontal locomotor activity (see Fig. 4g) in the open-field test. On the other hand, the female offspring were less responsive, as LNG treatment only slightly induced autism-like behavior, while LNG/EE and EE treatment showed little effect. Our results indicate that male offspring are more sensitive to prenatal levonorgestrel exposure-induced autism-like behavior. The detailed statistical information is shown below:

In Fig. 4a, two-way ANOVA revealed significant effect of sex [*F* (1, 56) = 4.594, *P* = 0.031] and a significant effect on treatment [*F* (3, 56) = 5.918, *P* < 0.01], and there was a significant interaction [*F* (3, 56) = 4.110, *P* = 0.042]. Subsequent post hoc analysis revealed that LNG and LNG/EE decreased buried marbles in male offspring (−50%, −40% vs. VEH group, respectively, *p* < 0.01), and the female offspring had significant less response in VEH and EE treatments compared to male offspring (*p* < 0.01).

In Fig. 4b, two-way ANOVA revealed significant effect of sex [*F* (1, 56) = 3.849, *P* = 0.041] and a significant effect on treatment [*F* (3, 56) = 5.101, *P* = 0.019], and there was a significant interaction [*F* (3, 56) = 3.917, *P* = 0.027]. Subsequent post hoc analysis revealed that LNG and LNG/EE decreased interaction time in male offspring (−26%, −33% vs. VEH group, respectively, *p* < 0.05); LNG decreased interaction time in female offspring (−35% vs. VEH group, *p* < 0.01), and the female offspring had significant less response in LNG/EE treatment compared to the male offspring (*p* < 0.01).

In Fig. 4c, two-way ANOVA revealed no significant effect of sex [*F* (1, 56) = 2.194, *P* = 0.061] and a significant effect on treatment [*F* (3, 56) = 3.691, *P* = 0.037], no significant interaction [*F* (3, 56) = 2.114, *P* = 0.167]. Subsequent post hoc analysis revealed that LNG and LNG/EE decreased time in open arm in male offspring (−22%, −16% vs. VEH group, respectively, *p* < 0.05), and LNG decreased time in open arm in female offspring (−15% vs. VEH group, *p* < 0.05).

In Fig. 4d, two-way ANOVA revealed significant effect of sex [*F* (1, 56) = 4.195, *P* = 0.023] and a significant effect on treatment [*F* (3, 56) = 6.348, *P* < 0.01], and there was a significant interaction [*F* (3, 56) = 3.987, *P* = 0.023]. Subsequent post hoc analysis revealed that LNG and LNG/EE increased time in closed arm in male offspring (+186%, +164% vs. VEH group, respectively, *p* < 0.05); and LNG increased time in closed arm in female offspring (+146% vs. VEH group, *p* < 0.05), and the female offspring had significant less response in LNG/EE treatment compared to male offspring (*p* < 0.05).

In Fig. 4e, two-way ANOVA revealed significant effect of sex [*F* (1, 64) = 3.447, *P* = 0.042] and a significant effect on treatment [*F* (3, 64) = 3.915, *P* = 0.036], and there was no significant interaction [*F* (3, 64) = 2.138, *P* = 0.194]. Subsequent post hoc analysis revealed that LNG and LNG/EE decreased central time in male offspring (−17%, −27% vs. VEH group, respectively, *p* < 0.05); LNG decreased central time in female offspring (−21% vs. VEH group, *p* < 0.05), and the female offspring had significant less response in LNG/EE treatment compared to male offspring (*p* < 0.05).

In Fig. 4f, two-way ANOVA revealed significant effect of sex [*F* (1, 64) = 3.461, *P* = 0.043] and a significant effect on treatment [*F* (3, 64) = 3.612, *P* = 0.037], and there was a significant interaction [*F* (3, 64) = 4.016, *P* = 0.031]. Subsequent post hoc analysis revealed that LNG and LNG/EE increased peripheral time in male offspring (+13%, +9% vs. VEH group, respectively, *p* < 0.05); LNG increased peripheral time in female offspring (+11% vs. VEH group, *p* < 0.05), and the female offspring had significant less response in LNG/EE treatment compared to male offspring (*p* < 0.05).

In Fig. 4g, two-way ANOVA revealed no significant effect of sex [*F* (1, 64) = 1.021, *P* = 0.561] and no significant effect on treatment [*F* (3, 64) = 1.014, *P* = 0.947] and no interaction [*F* (3, 64) = 0.948, *P* = 1.041].

### Overexpression of ERβ in the amygdala restores prenatal levonorgestrel exposure-induced ROS generation and DNA damage, along with dysfunction of mitochondria and fatty acid metabolism in 10-week-old offspring, while ERβ knockdown mimics the effect

**Fig. 5:**
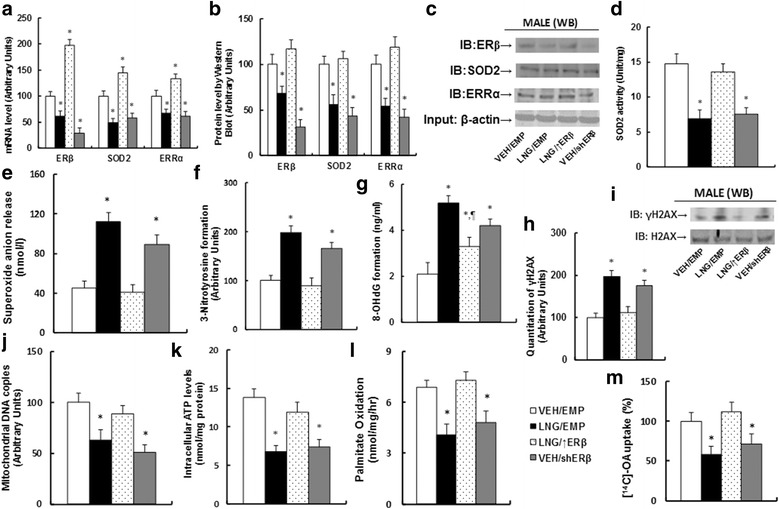
Overexpression of ERβ in the amygdala restores prenatal levonorgestrel exposure-induced ROS generation and DNA damage, along with dysfunction of mitochondria and fatty acid metabolism in 10-week-old offspring, while ERβ knockdown mimics the effect. The 8-week-old male offspring from the VEH group received either empty control (VEH/EMP) or ERβ knockdown (VEH/shERβ) lentivirus infusion, and the male offspring from the LNG group received either empty control (LNG/EMP) or ERβ expression (LNG/↑ERβ) lentivirus infusion, and the offspring were sacrificed at 10 weeks of age. **a**–**l** The amygdala tissues were isolated from 10-week-old treated male offspring for further analysis. **a** The mRNA levels for gene expression, *n* = 4. **b** The quantitation of protein levels, *n* = 5. **c** Representative bands for western blots. **d** SOD2 activity, *n* = 5. **e** In vivo superoxide anion release, *n* = 6. **f** Quantitation of 3-nitrotyrosine (3-NT) formation, *n* = 5. **g** 8-OHdG formation, *n* = 5. **h** Quantitation of γH2AX formation, *n* = 5. **i** Representative γH2AX western blotting band. **j** Mitochondrial DNA copies, *n* = 4. **k** Intracellular ATP levels, *n* = 5. **l** The in vivo palmitate oxidation rate, *n* = 5. **m** The amygdala neurons were isolated from 10-week-old treated male offspring for in vitro ^14^C–OA fatty acid uptake, *n* = 5. *P < 0.05 vs. VEH group; *P* < 0.05 vs. LNG group. Results are expressed as mean ± SEM

The 8-week-old male offspring from dams that were treated by either VEH or LNG received either EMP, ERβ overexpression (↑ERβ), or knockdown (shERβ) lentivirus infusion to the amygdala, and the offspring were sacrificed at 10 weeks of age for analysis of gene expression and the subsequent molecular consequences. Our results showed that the gene expression of ERβ and its target genes SOD2 and ERRα, including mRNA (see Fig. 5a) and protein levels (see Fig. 5b, c), decreased significantly in LNG/EMP treatment compared to that in the VEH/EMP group, and the expression increased in the LNG/↑ERβ group but decreased in the VEH/shERβ group. We also measured SOD2 activity (see Fig. 5d), and the results showed that LNG/EMP and VEH/shERβ treatment decreased SOD2 activity, while LNG/↑ERβ group increased SOD2 activity. Our results indicate that the manipulation of ERβ gene expression in the amygdala through lentivirus infusion was successful and efficient. We then measured the molecular consequences from the male offspring treated by ERβ lentivirus. Our results showed that LNG/EMP treatment significantly increased ROS formation, including superoxide anion release (see Fig. 5e), 3-nitrotyrosine formation (see Fig. 5f), and DNA damage, including 8-OHdG formation (see Fig. 5g) and γH2AX formation (see Fig. 5h, i). Overexpression of ERβ (LNG/↑ERβ group) completely normalized this effect compared to the VEH/EMP control group, while ERβ knockdown (VEH/shERβ group) mimicked the LNG effect. We then measured the function of mitochondria and fatty acid metabolism. Our results showed that LNG treatment (LNG/EMP group) significantly decreased mitochondrial DNA copies (see Fig. 5j), intracellular ATP levels (see Fig. 5k), in vivo palmitate oxidation rate (see Fig. 5l), and in vitro ^14^C–OA fatty acid uptake (see Fig. 5m) compared to the VEH/EMP control group. Again, ERβ expression (LNG/↑ERβ group) completely normalized the effect, and ERβ knockdown (VEH/shERβ group) mimicked the effect (see detailed statistical information in Additional file 1: Data S4). Our results indicate that ERβ expression in the amygdala plays a major role in prenatal levonorgestrel exposure-mediated molecular dysfunction in offspring.

### Overexpression of ERβ in the amygdala restores prenatal levonorgestrel exposure-induced autism-like behavior in 10-week-old offspring, while ERβ knockdown mimics the effect

**Fig. 6:**
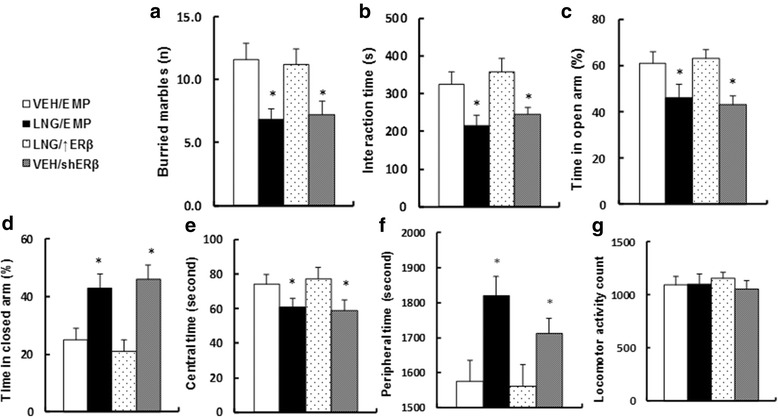
Overexpression of ERβ in the amygdala restores prenatal levonorgestrel exposure-induced autism-like behavior in 10-week-old offspring, while ERβ knockdown mimics the effect. The 8-week-old male offspring from the VEH group received either empty control (VEH/EMP) or ERβ knockdown (VEH/shERβ) lentivirus infusion, and the male offspring from the LNG group received either empty control (LNG/EMP) or ERβ expression (LNG/↑ERβ) lentivirus infusion. At 10 weeks of age, the offspring were used for autism-like behavior testing. **a** Buried marbles tests, *n* = 8. **b** Interaction time, *n* = 8. **c**, **d** Time spent in **c** open and **d** closed arms in the EPM test, *n* = 9. **e**–**g** Time spent in **e** central and **f** peripheral area and **g** locomotor activity in the open-field test, *n* = 9. *P < 0.05 vs. VEH group. Results are expressed as mean ± SEM

We measured the effect of ERβ expression on prenatal levonorgestrel exposure-induced autism-like behavior in offspring. The male offspring from the LNG/EMP treatment had significant less buried marbles (see Fig. 6a) and interaction time (see Fig. 6b) and spent significantly less time in the open arm (see Fig. 6c) and more time in the closed arm (see Fig. 6d) in the EPM test compared to that in the VEH/EMP control group. The offspring also spent less time in the central area (see Fig. 6e) and more time in the peripheral area (see Fig. 6f), but without changes in total horizontal locomotor activity (see Fig. 6g) in the open-field test. Overexpression of ERβ (LNG/↑ERβ group) completely normalized the effect, while ERβ knockdown (VEH/shERβ) mimicked it. Our results indicate that prenatal levonorgestrel exposure-induced autism-like behavior in offspring is due to ERβ suppression in the amygdala. The detailed statistical information is shown below:

In Fig. 6a, for buried marbles, one-way ANOVA revealed a significant effect [*F* (3, 31) = 8.162, *P* < 0.01]. Subsequent Tukey analysis revealed that LNG decreased buried marbles (−59% vs. VEH group, *p* < 0.01); ↑ERβ increased (+181% vs. EMP group, *p* < 0.01), while shERβ decreased (−38% vs. EMP group, *p* < 0.01) buried marbles.

In Fig. 6b, for interaction time, one-way ANOVA revealed a significant effect [*F* (3, 31) = 7.694, *P* < 0.01]. Subsequent Tukey analysis revealed that LNG decreased interaction time (−34% vs. VEH group, *p* < 0.01); ↑ERβ increased (+174% vs. EMP group, *p* < 0.01), while shERβ decreased (−25% vs. EMP group, *p* = 0.024) interaction time.

In Fig. 6c, for time in open arm, one-way ANOVA revealed a significant effect [*F* (3, 35) = 4.367, *P* = 0.032]. Subsequent Tukey analysis revealed that LNG decreased time in open arm (−25% vs. VEH group, *p* = 0.021); ↑ERβ increased (+137% vs. EMP group, *p* < 0.01), while shERβ decreased (−30% vs. EMP group, *p* = 0.024) time in open arm.

In Fig. 6d, for time in closed arm, one-way ANOVA revealed a significant effect [*F* (3, 35) = 8.127, *P* < 0.01]. Subsequent Tukey analysis revealed that LNG increased time in closed arm (+172% vs. VEH group, *p* < 0.01); ↑ERβ decreased (−52% vs. EMP group, *p* < 0.01), while shERβ increased (+187% vs. EMP group, *p* < 0.01) time in closed arm.

In Fig. 6e, for central time, one-way ANOVA revealed a significant effect [*F* (3, 35) = 4.361, *P* = 0.037]. Subsequent Tukey analysis revealed that LNG decreased central time (−18% vs. VEH group, *p* = 0.036); ↑ERβ increased (+126% vs. EMP group, *p* = 0.039), while shERβ decreased (−21% vs. EMP group, *p* = 0.037) central time.

In Fig. 6f, for peripheral time, one-way ANOVA revealed a significant effect [*F* (3, 35) = 3.794, *P* = 0.044]. Subsequent Tukey analysis revealed that LNG increased peripheral time (+115% vs. VEH group, *p* = 0.034); ↑ERβ decreased (−14% vs. EMP group, *p* = 0.041), while shERβ increased (+109% vs. EMP group, *p* = 0.043) peripheral time.

In Fig. 6g, for locomotor activity, one-way ANOVA revealed no significant effect [*F* (3, 35) = 0.594, *P* = 0.791].

## Discussion

In this study, we show that the compound LNG used in COC modifies the fetal condition during the prenatal period through altered methylation with ERβ suppression in the amygdala. ERβ suppression results in decreased expression of SOD2 and ERRα, and subsequently triggers damage in amygdala tissue through oxidative stress and the dysfunction of mitochondria and fatty acid metabolism, and eventually contributes to autism-like behavior in offspring.

The amygdala is responsible for storing memories of events for future recognition. It assists in the development of memories, fear emotion, and plays a major role in pleasure and sexual arousal, and many reports have shown that the amygdala is involved in ASD symptoms [37–39]. The functional significance of the changes only localized to the amygdala is social fear and/or anxiety learning [24, 40]. Our results show that prenatal LNG exposure induces ERβ suppression in the amygdala with autism-like behavior in offspring [12], and overexpression of ERβ completely restores this effect. This indicates that ERβ suppression in the amygdala may be a causative factor for autism-like behavior development. Furthermore, suppression of ERβ and its target genes, including SOD2 and ERRα, results in oxidative stress [41] and dysfunction of mitochondria and fatty acid metabolism [42–44], which may directly or indirectly contribute to tissue damage and the subsequent autism-like behavior development.

### Association of oral contraceptives and autism

The increase in the prevalence of oral contraceptive use in the past 60 years coincides with the recent dramatic rise in autism and ASD prevalence [8], indicating a link between oral contraceptive and ASD. The incidence of mild to moderate depressive syndrome among women taking oral contraceptives is ~10 to 40% [45], and several studies demonstrated that oral contraceptives have a negative effect on anxiety, mood, and emotional well-being in women [46–49]. It has been reported that long-term administration of combined LNG and EE, two of the most widely used steroids in oral contraceptive pills, is associated with a reduction in social behavior [50]. It is reasonable that the neurochemical changes induced by LNG/EE treatment may contribute to some of the emotional disorders observed in hormonal contraceptive users. The combined oral contraceptive, especially LNG, is used by women to prevent pregnancy after birth control failure. This medication is a progestin hormone that works primarily by preventing the release of an egg (ovulation) during the menstrual cycle. The suppression of ovulation produced by oral contraceptives is an indisputable abnormality [8], while to date, there is no comprehensive research on the potential neurodevelopmental effects of oral contraceptive use on oocyte and progeny. Our study shows that prenatal LNG exposure induces ERβ suppression in the amygdala in offspring with autism-like behavior, which includes significantly less buried marbles and interaction time in the social behavior test and spending much less time in the open arm and more time in the closed arm in the EPM test, as well as spending less time in the central area and more time in the peripheral area in the open-field test. This indicates that prenatal exposure to oral contraceptives may be a potential risk factor for ASD or related neurodevelopment abnormalities in offspring. On the other hand, we must emphasize that because of the high doses of the hormones administered, as well as the long duration of time, the applicability of these findings to the human condition is limited.

### ERβ suppression may contribute to male predominance in autism

ASD is much more common in males than in females, with a ratio of 4:1. It has been reported that high levels of testosterone during early fetal development may be a risk factor for ASD. This is supported by many studies reporting an association between fetal testosterone levels and autistic features [24, 51, 52], while the related mechanism is still unknown. ERβ is largely expressed in brain tissues [53], and it has been recently reported that there are alterations in ERβ expression in the brain of ASD subjects [14]. ERβ-deficient mice also show increased anxiety [13], indicating that ERβ may play an important role in ASD development [54]. Our results show that prenatal exposure of EE has no effect on autism-like behavior, which is consistent with previous observations that estrogen is a neuroprotective hormone [55]. On the other hand, prenatal exposure to oral contraceptive LNG induces ERβ suppression with autism-like behavior in offspring. Furthermore, we show that male offspring are more susceptible to LNG-induced ERβ suppression with higher autism-like behavior ratio. This may be due to the following reasons: (a) higher endogenous estrogen levels in female offspring partly normalize the testosterone effect of LNG; (b) the female offspring have a higher basal expression of ERβ compared to male offspring, which is more resistant to LNG-induced ERβ suppression; and (c) higher estrogen levels in female offspring could activate ERβ and upregulate its target genes SOD2 and ERRα and subsequently ameliorate tissue damage [15, 16] with less responsiveness to autism-like behavior development. In this study, the LNG/EE treatment induces effects like those of LNG in many of the biochemical and behavioral parameters examined, while sometimes in female offspring, the LNG/EE shows an effect to that of EE instead of LNG alone. This may be due to that the potential effect of LNG is partly normalized or antagonized by estrogen.

### Potential oral contraceptive exposure from the environment

Increasing evidence shows that the environment also plays an important role in the etiology of ASD [4]. It has been reported that the concordance rate for ASD in dizygotic twins is around 30% [56], which is much higher than the 8–17% concordance rate for non-twin siblings [57], indicating that environmental factors significantly contribute to ASD. Recently, specific environmental factors, including prenatal and perinatal exposure factors, have been reported to contribute to increased ASD risk [24, 58]. In our study, prenatal LNG exposure in dams during pregnancy induces autism-like behavior in offspring, indicating that oral contraceptive exposure may be an important environmental factor in ASD development. Currently in China, other than the intentional uptake of oral contraceptive pills for birth control, another important increasing source of prenatal LNG exposure comes from seafood contaminated with COC. COC has been widely used in China in the seafood industry to prevent the pregnancy of female organisms, such as fish and shrimp, so that they can grow more quickly and become fatter without pregnancy. This may be an important reason for why there is continuously increasing incidence of ASD in China. Pregnant women must be especially careful to avoid eating oral contraceptive-contaminated seafood.

## Conclusions

Altogether, we show that prenatal exposure to oral contraceptive LNG alters DNA methylation on the ERβ promoter with ERβ suppression in the amygdala. This subsequently results in tissue damage with oxidative stress and dysfunction of mitochondria and fatty acid metabolism and triggers autism-like behavior in offspring. Meanwhile, EE seems to have no effect, and male offspring are more susceptible than female offspring. Overexpression of ERβ restores LNG-mediated effects, while ERβ knockdown mimics the effect. This is the first time the potential effect of prenatal exposure to oral contraceptives on the contribution of autism-like behavior in offspring is discovered.

## Additional file

Additional file 1: Table S1. Sequences of primers for the real-time quantitative PCR (qPCR).**Table S2.** mRNA level by qPCR in the hypothalamus, hippocampus, and amygdala from 10 weeks old offspring. **Figure S1.** Prenatal levonorgestrel exposure does not affect the expression of ERβ and its target genes in the hypothalamus and hippocampus in 10-week-old offspring. Three-month-old pregnant dams were exposed to LNG (20 μg levonorgestrel), EE (10 μg ethinyl estradiol), LNG/EE (20 μg LNG and 10 μg EE combination), or VEH (vehicle, 5% ethanol in organic sesame oil) by subcutaneous daily injection of 0.1 ml for 21 days until pup delivery. Both male and female offspring were sacrificed at 10 weeks old to isolate the hypothalamus and hippocampus tissues for further analysis. (a–c) The mRNA levels in the hypothalamus for genes of ERβ (a), SOD2 (b), and ERRα (c), *n* = 5. (d–f) The mRNA levels in the hippocampus for genes of ERβ (d), SOD2 (e), and ERRα (f), *n* = 5. Results are expressed as mean ± SEM. Data S1 Statistical details for Fig. 1. Data S2 Statistical details for Fig. 2. Data S3 Statistical details for Fig. 3. Data S4 Statistical details for Fig. 5. (DOCX 63 kb)

## Abbreviations

AR: Androgen receptor
ASD: Autism spectrum disorder
ChIP: Chromatin immunoprecipitation
COC: Combined oral contraceptives
EE: Ethinyl estradiol
EMP: Empty control
EPM: Elevated plus maze
ERR: Estrogen-related receptor α
ERα: Estrogen receptor α
ERβ: Estrogen receptor β
GPER1: G protein-coupled estrogen receptor-1
LNG: Levonorgestrel
MBT: Marbles burying test
SI: Social interaction
SOD2: Mitochondrial superoxide dismutase
VEH: Vehicle control
