# Supplementary material for: Prenatal levonorgestrel exposure induces autism-like behavior in offspring through ERβ suppression in the amygdala
Source: Mol Autism. 2017 Aug 17;8:46. doi: 10.1186/s13229-017-0159-3 (PMC5561609; doi:10.1186/s13229-017-0159-3)
Supplement: Additional file 1: Table S1. — Sequences of primers for the real-time quantitative PCR (qPCR).Table S2. mRNA level by qPCR in the hypothalamus, hippocampus, and amygdala from 10 weeks old offspring. Figure S1. Prenatal levonorgestrel exposure does not affect the expression of ERβ and its target genes in the hypothalamus and hippocampus in 10-week-old offspring. Three-month-old pregnant dams were exposed to LNG (20 μg levonorgestrel), EE (10 μg ethinyl estradiol), LNG/EE (20 μg LNG and 10 μg EE combination), or VEH (vehicle, 5% ethanol in organic sesame oil) by subcutaneous daily injection of 0.1 ml for 21 days until pup delivery. Both male and female offspring were sacrificed at 10 weeks old to isolate the hypothalamus and hippocampus tissues for further analysis. (a–c) The mRNA levels in the hypothalamus for genes of ERβ (a), SOD2 (b), and ERRα (c), n = 5. (d–f) The mRNA levels in the hippocampus for genes of ERβ (d), SOD2 (e), and ERRα (f), n = 5. Results are expressed as mean ± SEM. Data S1 Statistical details for Fig. 1. Data S2 Statistical details for Fig. 2. Data S3 Statistical details for Fig. 3. Data S4 Statistical details for Fig. 5. (DOCX 63 kb) [file 13229_2017_159_MOESM1_ESM.docx]

**Prenatal Levonorgestrel Exposure Induces Autism-Like Behavior in Offspring through ERβ Suppression in the Amygdala**

**Supplemental Data**

**Table S1. Sequences of primers for the real timequantitative PCR (qPCR)**

| Gene | Species | Forward primer (5'→3') | Reverse primer (5'→3') |
| --- | --- | --- | --- |
| AR | Rat | aacaaccagcctgattccttt | gaatgactgccatctggtcat |
| β-actin | Rat | ttccttcctgggtatggaatc | cttctgcatcctgtcagcaat |
| ERα | Rat | tcaatgatgggcttattgacc | gaccaatcatcaggatctcca |
| ERβ | Rat | tcagcatgaagtgcaaaaatg | ggttctgggagctctctttgt |
| ERRα | Rat | cagtgggaagctagtgctcag | ggacagctgtactcgatgctc |
| GPER1 | Rat | cttggtggtgaacatcagctt | gagcacggcgatatcgtaata |
| SOD2 | Rat | caactcaggttgctcttcagc | ctcaaaagacccaaagtcacg |

**Table S2. mRNA level by qPCR in the hypothalamus, hippocampus and amygdala from 10 weeks old offspring**

| Genes/  Expression | **Male** | | | **Female** | | |
| --- | --- | --- | --- | --- | --- | --- |
|  | hypothalamus | hippocampus | amygdala | hypothalamus | hippocampus | amygdala |
| AR | 100 ± 11 | 87 ± 9 | 103 ± 12 | 94 ± 7 | 113 ± 9 | 104 ± 6 |
| ERα | 89 ± 12 | 111 ± 12 | 103 ± 9 | 115 ± 14 | 103 ± 10 | 91 ± 15 |
| GPER1 | 96 ± 8 | 94 ± 13 | 102 ± 7 | 109 ± 11 | 96 ± 14 | 101 ± 11 |

**Figure S1**

**Figure S1. Prenatal levonorgestrel exposure does not affect the expression of ERβ and its target genes in the hypothalamus and hippocampus in 10-week old offspring.** 3-month old pregnant dams were exposed to LNG (20μg levonorgestrel), EE (10μg ethynyl estradiol), LNG/EE (20μg LNG and 10μg EE combination), or VEH (vehicle, 5% ethanol in organic sesame oil) by subcutaneous daily injection of 0.1ml for 21 days until pup delivery. Both male and female offspring were sacrificed at 10 weeks old to isolate the hypothalamus and hippocampus tissues for further analysis. (a-c) The mRNA levels in hypothalamus for genes of ERβ (a), SOD2 (b) and ERRα (c), n=5. (d-f) The mRNA levels in hippocampus for genes of ERβ (d), SOD2 (e) and ERRα (f), n=5. Results are expressed as mean ± SEM.

**Data S1. Statistical details for Figure 1**

In Fig 1a, two-way ANOVA revealed a significant effect of sex, [F(1, 32)=4.698, P=0.038], and a significant effect on treatment [F(3,32)=5.765, P=0.021], while there was significant interaction [F(3,32)= 3.735, P=0.042]. Subsequent post hoc analysis revealed that LNG and LNG/EE in male decreased ERβ expression (-55%, -27% vs. VEH group, respectively, p<0.01), and in female, LNG decreased ERβ expression (-28% vs. VEH group, p=0.023); and the female had significant less response in LNG treatment compared to male (p=0.034).

In Fig 1b, two-way ANOVA revealed a significant effect of sex, [F(1, 32)=4.124, P=0.045], and a significant effect on treatment [F(3,32)=5.121, P=0.031], while there was no significant interaction [F(3,32)= 2.341, P=0.145]. Subsequent post hoc analysis revealed that LNG and LNG/EE in male decreased ERβ expression (-44%, -21% vs. VEH group, respectively, p<0.01), and in female, LNG decreased ERβ expression (-24% vs. VEH group, p=0.023); and the female had significant less response in LNG treatment compared to male (p=0.042).

In Fig 1c, two-way ANOVA revealed a significant effect of sex, [F(1, 32)=4.354, P=0.033], and a significant effect on treatment [F(3,32)=5.312, P=0.023], and there was significant interaction [F(3,32)=4.615, P=0.037]. Subsequent post hoc analysis revealed that LNG and LNG/EE in male decreased ERβ expression (-35%, -28% vs. VEH group, respectively, p<0.01); and the female had significant less response in LNG and LNG/EE treatments compared to male (p=0.014 and 0.006, respectively).

In Fig 1e, for male offspring, one-way ANOVA revealed a significant effect on ERβ [F(3,19)= 11.326, P<0.001]; a significant effect on SOD2 [F(3,19)=10.164, P<0.001] and a significant effect on ERRα [F(3,19)=9.419, P<0.001]. Subsequent Turkey analysis revealed that LNG and LNG/EE treatment decreased ERβ expression (-58%, -51% vs. VEH group, respectively, p<0.01); decreased SOD2 expression (-46%, -23% vs. VEH group, respectively, p<0.01); decreased ERRα expression (-32%, -25% vs. VEH group, respectively, p<0.01).

In Fig 1g for female offspring, one-way ANOVA revealed a significant effect on ERβ [F(3,19)= 4.251, P=0.034]; a significant effect on SOD2 [F(3,19)=4.651, P=0.025], and there was no significant effect on ERRα [F(3,19)=0.487, P=0.356]. Subsequent Turkey analysis revealed that LNG treatment decreased ERβ and SOD2 expression (-22%, -32% vs. VEH group, respectively, p<0.01).

In Fig 1h for SOD2 activity, two-way ANOVA revealed a significant effect of sex, [F(1, 40)=5.126, P=0.027], and a significant effect on treatment [F(3,40)=7.164, P<0.001], and there was significant interaction [F(3,40)=4.539, P=0.034]. Subsequent post hoc analysis revealed that LNG decreased SOD2 activity in male and female offspring (-57%, -24% vs. VEH group, respectively, p<0.01); LNG/EE decreased SOD activity in male offspring (-35% vs. VEH group, p<0.01); and the female offspring had significant less response in LNG and LNG/EE treatments compared to male (p<0.01).

**Data S2. Statistical details for Figure 2**

In Fig 2b for ERβ promoter methylation, two-way ANOVA revealed no significant effect of sex, [F(1, 32)=2.319, P=0.069], a significant effect on treatment [F(3,32)=14.615, P<0.001], and there was no significant interaction [F(3,32)=2.459, P=0.106]. Subsequent post hoc analysis revealed that LNG and LNG/EE increased methylation in male offspring (+184%, +194% vs. VEH group, respectively, p<0.001); and increased methylation in female offspring (+214%, +156% vs. VEH group, respectively, p<0.01); and in female offspring, LNG/EE had significant less response compared to LNG treatment (p<0.01).

In Fig 2c for male offspring, one-way ANOVA revealed a significant effect on H3K9me2 [F(3,19)=14.312, P<0.001], a significant effect on H3K27me3 [F(3,19)=15.389, P<0.001], and no significant effect on H3K9me3 [F(3,19)=1.318, P=0.196]. Subsequent Turkey analysis revealed that LNG and LNG/EE increased binding ability of H3k9me2 (+192%, +156% vs. VEH group, respectively, p<0.01); and H3K27me3 (+231%, +169% vs. VEH group, respectively, p<0.01); and in H3K27me3, LNG/EE had significant less response compared to LNG treatment (p<0.01).

In Fig 2d for female offspring, one-way ANOVA revealed a significant effect on H3K9me2 [F(3,19)=17.914, P<0.001], a significant effect on H3K27me3 [F(3,19)=14.614, P<0.001], and no significant effect on H3K9me3 [F(3,19)=1.367, P=0.124]. Subsequent Turkey analysis revealed that LNG and LNG/EE increased binding ability of H3k9me2 (+231%, +187% vs. VEH group, respectively, p<0.01); and H3K27me3 (+189%, +145% vs. VEH group, respectively, p<0.01); and in H3K27me3, LNG/EE had significant less response compared to LNG treatment (p<0.01).

**Data S3. Statistical details for Figure 3**

In Fig 3a, two-way ANOVA revealed significant effect of sex, [F(1, 40)=3.651, P=0.035], and a significant effect on treatment [F(3,40)=12.964, P<0.001], and significant interaction [F(3,40)=4.694, P=0.021]. Subsequent post hoc analysis revealed that LNG and LNG/EE increased superoxide anion release in male offspring (+284%, +211% vs. VEH group, respectively, p<0.01); and increased superoxide anion release in female offspring (+200%, +169% vs. VEH group, respectively, p<0.01); and the female offspring had significant less response in LNG and LNG/EE treatments compared to male offspring (p<0.01).

In Fig 3b, two-way ANOVA revealed significant effect of sex, [F(1, 32)=3.456, P=0.042], and a significant effect on treatment [F(3,32)=11.137, P<0.001], and significant interaction [F(3,32)= 4.310, P=0.032]. Subsequent post hoc analysis revealed that LNG and LNG/EE increased 3-nitrotyrosine formation in male offspring (+215%, +178% vs. VEH group, respectively, p<0.01); and increased 3-nitrotyrosine formation in female offspring (+165%, +145% vs. VEH group, respectively, p<0.01); and the female offspring had significant less response in LNG and LNG/EE treatments compared to male offspring (p<0.05).

In Fig 3c, two-way ANOVA revealed significant effect of sex, [F(1, 32)=3.126, P=0.043], and a significant effect on treatment [F(3,32)=10.108, P<0.001], and significant interaction [F(3,32)= 4.657, P=0.025]. Subsequent post hoc analysis revealed that LNG and LNG/EE increased 8-OHdG formation in male offspring (+253%, +221% vs. VEH group, respectively, p<0.001); and increased 8-OHdG formation in female offspring (+238%, +181% vs. VEH group, respectively, p<0.01); and the female offspring had significant less response in LNG and LNG/EE treatments compared to male offspring (p<0.05).

In Fig 3e, two-way ANOVA revealed significant effect of sex, [F(1, 32)=3.156, P=0.036], and a significant effect on treatment [F(3,32)=9.631, P<0.001], and significant interaction [F(3,32)=4.416, P=0.014]. Subsequent post hoc analysis revealed that LNG and LNG/EE increased γH2AX formation in male offspring (+216%, +189% vs. VEH group, respectively, p<0.001); and LNG increased γH2AX formation in female offspring (+154% vs. VEH group, p<0.01); and the female offspring had significant less response in LNG and LNG/EE treatments compared to male offspring (p<0.01).

In Fig 3f, two-way ANOVA revealed no significant effect of sex, [F(1, 24)=2.100, P=0.091], and a significant effect on treatment [F(3,24)=6.149, P<0.01], no significant interaction [F(3,24)= 3.146, P=0.114]. Subsequent post hoc analysis revealed that LNG and LNG/EE decreased mitochondrial DNA copies in male offspring (-43%, -22% vs. VEH group, respectively, p<0.01); and decreased mitochondrial DNA copies in female offspring (-26%, -21% vs. VEH group, respectively, p<0.05).

In Fig 3g, two-way ANOVA revealed no significant effect of sex, [F(1, 32)=2.569, P=0.132], and a significant effect on treatment [F(3,32)=9.234, P<0.001], no significant interaction [F(3,32)= 2.194, P=0.087]. Subsequent post hoc analysis revealed that LNG and LNG/EE decreased Intracellular ATP in male offspring (-50%, -44% vs. VEH group, respectively, p<0.01); and decreased Intracellular ATP in female offspring (-38%, -27% vs. VEH group, respectively, p<0.01); and the female offspring had significant less response in LNG and LNG/EE treatments compared to male offspring (p<0.05).

In Fig 3h, two-way ANOVA revealed significant effect of sex, [F(1, 32)=4.125, P=0.041], and a significant effect on treatment [F(3,32)=7.915, P<0.01], there was significant interaction [F(3,32)= 4.603, P=0.024]. Subsequent post hoc analysis revealed that LNG and LNG/EE decreased palmitate oxidation in male offspring (-42%, -18% vs. VEH group, respectively, p<0.05); and LNG decreased palmitate oxidation in female offspring (-19% vs. VEH group, p<0.05); and the female offspring had significant less response in LNG and LNG/EE treatments compared to male offspring (p<0.05).

In Fig 3i, two-way ANOVA revealed significant effect of sex, [F(1, 32)=4.247, P=0.037], and a significant effect on treatment [F(3,32)=6.371, P<0.01], there was significant interaction [F(3,32)=4.119, P=0.039]. Subsequent post hoc analysis revealed that LNG and LNG/EE decreased fatty acid uptake in male offspring (-33%, -41% vs. VEH group, respectively, p<0.05); and LNG decreased fatty acid uptake in female offspring (-23% vs. VEH group, p<0.05); and the female offspring had significant less response in LNG/EE treatments compared to male offspring (p<0.05).

**Data S4. Statistical details for Figure 5**

In Fig 5a for mRNA expression, regarding the ERβ, one-way ANOVA revealed a significant effect [F(3,15)= 11.461, P<0.01]. Subsequent Turkey analysis revealed that LNG decreased ERβ expression (-39% vs. VEH group, p<0.01); and ↑ERβ increased (+311% vs. EMP group, p<0.001), while shERβ decreased (-67% vs. EMP group, p<0.01) ERβ expression. Regarding the SOD2, one-way ANOVA revealed a significant effect [F(3,15)=10.367, P<0.001], Subsequent Turkey analysis revealed that LNG decreased SOD2 expression (-51% vs. VEH group, p<0.01); and ↑ERβ increased (+287% vs. EMP group, p<0.001), while shERβ decreased (-42% vs. EMP group, p<0.001) SOD2 expression. Regarding the ERRα, one-way ANOVA revealed a significant effect [F(3,15)= 9.165, P<0.001]. Subsequent Turkey analysis revealed that LNG decreased ERRα expression (-33% vs. VEH group, p<0.01); and ↑ERβ increased (+200% vs. EMP group, p<0.001), while shERβ decreased (-39% vs. EMP group, p<0.01) ERRα expression.

In Fig 5b for protein expression, regarding the ERβ, one-way ANOVA revealed a significant effect [F(3,19)=7.254, P<0.01]. Subsequent Turkey analysis revealed that LNG decreased ERβ expression (-32% vs. VEH group, p<0.01); and ↑ERβ increased (+188% vs. EMP group, p<0.01), while shERβ decreased (-69% vs. EMP group, p<0.001) ERβ expression. Regarding the SOD2, one-way ANOVA revealed a significant effect [F(3,19)= 8.997, P<0.01]. Subsequent Turkey analysis revealed that LNG decreased SOD2 expression (-44% vs. VEH group, p<0.01); and ↑ERβ increased (+184% vs. EMP group, p<0.01), while shERβ decreased (-57% vs. EMP group, p<0.01) SOD2 expression. Regarding the ERRα, one-way ANOVA revealed a significant effect [F(3,19)= 7.316, P<0.01]. Subsequent Turkey analysis revealed that LNG decreased ERRα expression (-46% vs. VEH group, p<0.01); and ↑ERβ increased (+217% vs. EMP group, p<0.01), while shERβ decreased (-58% vs. EMP group, p<0.01) ERRα expression.

In Fig 5d for SOD2 activity, one-way ANOVA revealed a significant [F(3,19)= 8.716, P<0.01]. Subsequent Turkey analysis revealed that LNG decreased SOD2 activity (-53% vs. VEH group, p<0.01); and ↑ERβ increased (+194% vs. EMP group, p<0.01), while shERβ decreased (-49% vs. EMP group, p<0.001) SOD2 activity.

In Fig 5e for superoxide anion release, one-way ANOVA revealed a significant effect [F(3,23)= 9.678, P<0.001]. Subsequent Turkey analysis revealed that LNG increased superoxide anion release (+221% vs. VEH group, p<0.01); and ↑ERβ decreased (-63% vs. EMP group, p<0.01), while shERβ increased (+187% vs. EMP group, p<0.01) superoxide anion release.

In Fig 5f for 3-nitrotyrosine formation, one-way ANOVA revealed a significant effect [F(3,19)= 8.419, P<0.001]. Subsequent Turkey analysis revealed that LNG increased 3-nitrotyrosine formation (+198% vs. VEH group, p<0.01); and ↑ERβ decreased (-55% vs. EMP group, p<0.01), while shERβ increased (+165% vs. EMP group, p<0.01) 3-nitrotyrosine formation.

In Fig 5g for 8-OHdG formation, one-way ANOVA revealed a significant effect [F(3,19)= 10.319, P<0.001]. Subsequent Turkey analysis revealed that LNG increased 8-OHdG formation (+248% vs. VEH group, p<0.01); and ↑ERβ decreased (-37% vs. EMP group, p<0.01), while shERβ increased (+201% vs. EMP group, p<0.01) 8-OHdG formation.

In Fig 5h for γH2AX formation, one-way ANOVA revealed a significant effect [F(3,19)= 8.769, P<0.01]. Subsequent Turkey analysis revealed that LNG increased γH2AX formation (+198% vs. VEH group, p<0.01); and ↑ERβ decreased (-43% vs. EMP group, p<0.01), while shERβ increased (+176% vs. EMP group, p<0.01) γH2AX formation.

In Fig 5j for mitochondrial DNA copies, one-way ANOVA revealed a significant effect [F(3,15)= 6.164, P<0.01]. Subsequent Turkey analysis revealed that LNG decreased mitochondrial DNA copies (-37% vs. VEH group, p=0.034); and ↑ERβ increased (+141% vs. EMP group, p=0.036), while shERβ decreased (-48% vs. EMP group, p<0.01) mitochondrial DNA copies.

In Fig 5k for intracellular ATP levels, one-way ANOVA revealed a significant effect [F(3,19)= 10.541, P<0.001]. Subsequent Turkey analysis revealed that LNG decreased intracellular ATP levels (-51% vs. VEH group, p<0.01); and ↑ERβ increased (+182% vs. EMP group, p<0.01), while shERβ decreased (-46% vs. EMP group, p<0.01) intracellular ATP levels.

In Fig 5l for palmitate oxidation, one-way ANOVA revealed a significant effect [F(3,19)=9.124, P<0.001]. Subsequent Turkey analysis revealed that LNG decreased palmitate oxidation (-59% vs. VEH group, p<0.01); and ↑ERβ increased (+175% vs. EMP group, p<0.01), while shERβ decreased (-31% vs. EMP group, p<0.01) palmitate oxidation.

In Fig 5m for [^14^C]-OA uptake, one-way ANOVA revealed a significant effect [F(3,19)= 7.619, P<0.01]. Subsequent Turkey analysis revealed that LNG decreased [^14^C]-OA uptake (-42% vs. VEH group, p<0.01); and ↑ERβ increased (+197% vs. EMP group, p<0.01), while shERβ decreased (-29% vs. EMP group, p=0.036) [^14^C]-OA uptake.
